# Copper, Manganese, Selenium and Zinc in Wild-Growing Edible Mushrooms from the Eastern Territory of “Green Lungs of Poland”: Nutritional and Toxicological Implications

**DOI:** 10.3390/ijerph16193614

**Published:** 2019-09-26

**Authors:** Iwona Mirończuk-Chodakowska, Katarzyna Socha, Małgorzata Elżbieta Zujko, Katarzyna Maria Terlikowska, Maria Halina Borawska, Anna Maria Witkowska

**Affiliations:** 1 Department of Food Biotechnology, Faculty of Health Sciences, Medical University of Bialystok, Szpitalna 37, 15-295 Bialystok, Poland; malgorzata.zujko@umb.edu.pl (M.E.Z.); katarzyna.terlikowska@umb.edu.pl (K.M.T.); witam@umb.edu.pl (A.M.W.); 2 Department of Bromatology, Faculty of Pharmacy with the Division of Laboratory Medicine, Mickiewicza 2D, 15-222 Bialystok, Poland; katarzyna.socha@umb.edu.pl (K.S.); borawska@umb.edu.pl (M.H.B.)

**Keywords:** mushrooms, food, intake, copper, manganese, selenium, zinc

## Abstract

The aims of this study were to determine Cu, Mn, Se, and Zn content in wild mushrooms collected from unpolluted areas of the eastern Green Lungs of Poland (GLP) territory, to compare them to some popular species of cultivated mushrooms, evaluate mushroom contribution to the daily intake of the studied bioelements, and to determine their possible toxic effect resulting from potentially excessive mushroom consumption from areas recognized as ecologically uncontaminated. Bioelements were determined in 21 species of edible mushrooms: eighteen species of wild mushrooms and three species of popular cultivated mushrooms. The mean Cu, Mn, Se, and Zn content (in µg/g, dry mass DM) ranged from 10.6–123.1, 12.2–41, 0.13–13.3, and 68.3–184, respectively. A comparison with recommended dietary allowance (RDA) for Cu, Se, and Zn as well as adequate intake (AI) for Mn demonstrated that a 100 g fresh mass (FM) portion of mushroom species with the highest content of a given element can meet the demand for Cu, Mn, Se and Zn at 203%, 14–17%, 211%, and 16–22%, respectively. A comparison of the content of the examined bioelements contained in one portion of mushrooms (100 g FM) against the toxicological intake limits for different chemical elements with the provisional maximum tolerable daily intake (PMTDI) and upper intake level (UL) showed no risk of toxicity for the evaluated mushroom species.

## 1. Introduction

The Green Lungs of Poland (GLP) is a unique natural area located within five voivodeships of northeastern Poland. It is among the least polluted areas in Poland, which is characterized by low levels of urbanization, low population density, the presence of large and compact nature-rich forest complexes, and the absence of heavy industry. In 1994, the Polish authorities recognized the GLP as a region where comprehensive nature conservation should be ensured and the idea of eco-development should be respected [1].

In regions of the world where the gathering and consumption of mushrooms are popular, they can be a significant source of various nutrients—carbohydrates and proteins [2,3], vitamins [4], minerals [5,6,7], and biologically active ingredients, with antioxidant and anti-inflammatory activity, such as phenols and ergosterol [8,9]. The mineral content in mushrooms is significant, and the proportions of elements are beneficial for health [10,11,12,13,14,15]. The fruiting bodies of mushrooms are characterized by low Na content [12] with a significant content of K [10,12], Ca, and Mg [10,12,14,15]. Such proportions of minerals are particularly beneficial for people with cardiovascular diseases [11,13,16,17].

Cu, Mn, Se, and Zn are elements that perform important biological functions in the human body. Cu and Zn are components of superoxide dismutase (SOD1), found mostly in the cytosol and SOD3 (extracellular), whereas Mn is a component of SOD2 (mitochondrial). Se is a part of two key amino acids, selenomietionine and selenocysteine, building key enzymes: glutathione peroxidase and thioredoxin reductase. In addition to the antioxidant function, Cu plays a key role in bone formation, iron metabolism, and heme synthesis, and determines the proper functioning of the nervous system. Mn is involved in numerous metabolic processes and is one of the factors conditioning their normal course. Se participates in thyroid hormone metabolism and plays an important role in proper immune system functioning, stimulating the production of T lymphocytes and their proper response. Zn is also responsible for the proper functioning of the nervous and endocrine systems, and it is particularly important for the hormones of the thyroid and pancreas [18,19,20,21,22]. 

The main source of elements is food. Their content in food products is conditioned mainly by the presence of elements in the soil and a number of factors favoring their uptake from the substrate by plants and mushrooms. In the case of mushrooms, the element content in fruiting bodies is determined on the one hand by environmental factors, such as substrate type and properties, organic matter content, pH, element content in the soil, and on the other hand by individual factors, such as species, morphological part, development stage, mycelium age, biochemical composition, and spaces between the fruiting bodies [23,24].

The element content in mushrooms (Cu, Mn, Se, and Zn) is significantly affected by environmental pollution, especially the presence of heavy metals such as Hg, Cd, and Pb, which may foster mutual interference with the elements and reduce their bioavailability [25,26,27,28,29]. Furthermore, mushrooms have a high ability to accumulate trace elements. The content of these elements in the fruiting bodies may be a determinant of environmental purity. [27,28,29]. Mushrooms show proven cumulative properties of both non-essential trace elements (Cd, Pb, Hg) as well as essential trace elements (Cu, Mn, Se, and Zn) [28,29].

Therefore, the aims of this study were to determine the content of wild mushrooms from the eastern GLP territory, compare them with Cu, Mn, Se, and Zn content in some popular species of cultivated mushrooms, evaluate mushroom contribution to the daily intake of the examined bioelements, and determine their possible toxic effect resulting from potentially excessive mushroom consumption from areas recognized as ecologically uncontaminated. 

According to our knowledge, this is the first study that presents, in summary, the content of Cu, Mn, Se, and Zn essential trace elements in 21 edible mushroom species. At the same time this study emphasizes the mineral nutritional value and analyzes the potential toxicological risk of mushroom consumption.

## 2. Materials and Methods

### 2.1. Studied Material

The material for the study consisted of 21 of the most popular species of edible mushrooms in Poland (3–4 samples of each species), including 18 species of wild-growing and 3 species of cultivated mushrooms for comparative purposes.

Each of the samples of one species was obtained from different locations so as not to originate from one mycelium. The other parameter for mushroom selection was their occurrence far from transportation routes and other sources of anthropogenic pollution. The fruiting bodies were fully developed (size typical of each species) and not infested with insects.

Cultivated mushrooms were purchased at the local market, with three to four samples from separate batches for each of the three species. In total, 68 mushroom samples were studied. 

Among the wild mushrooms, the following were obtained:

- included on the list of mushrooms authorized for trade in Poland: *Armillaria mellea* (Vahl) P. Kumm; *Boletus edulis* Bull.; *Boletus subtomentosus* (L.); *Cantharellus cibarius* Fr.; *Cortinarius caperatus* (Pers. Fr.); *Imleria badia* (Fr.) Fr.; *Lactarius deliciosus* (L.) Gray; *Leccinum aurantiacum* (Bull.) Gray; *Leccinum rufum* (Schaeff.) Kreisel; *Leccinum scabrum* (Bull.) Gray; *Macrolepiota procera* (Scop.) Singer; *Suillus bovinus* (Pers.) Roussel; *Suillus grevillei* (Klotzch) Singer; *Suillus luteus* (L.) Roussel; *Tricholoma equestre* (L.) P. Kumm.; *Tricholoma portentosum* (Fr.) Quel.; *Xerocomellus chrysenteron* (Bull.) Šutara.
- as well as recreationally picked (outside of the list of mushroom species authorized for sale): *Russula heterophylla* (Fr.) Fr.; *Russula vinosa* Lindblad. The fruiting bodies of the mushrooms were classified using atlases and identification keys [30,31].

For comparative purposes, these cultivated mushroom species available for sale at the local market were considered: *Agaricus bisporus* (J.E. Lange) Imbach; *Lentinula edodes* (Berk.) Singer.; *Pleurotus ostreatus* (Jacq.) P. Kumm; 

The mushroom samples were approximately 200–300 g. Then, undergrowth components (soil, leaves, insects, and others) were manually removed and the mushrooms were cut into smaller pieces. 

### 2.2. Physiographic Characteristic of the Study Area

**Figure 1:**
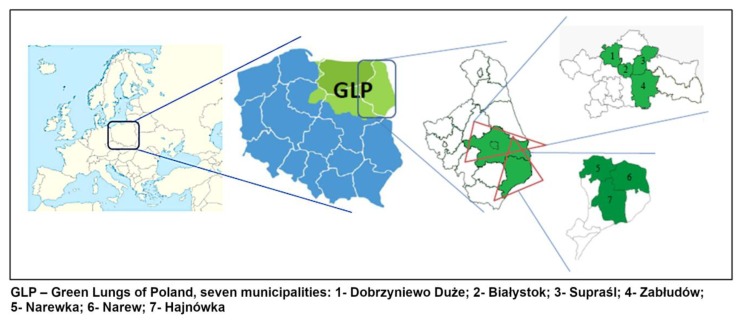
Location of the sampling area.

Wild-growing mushrooms came from an area of seven municipalities of the eastern part of the Green Lungs of Poland: Hajnówka, Narew, Narewka, which belong to Hajnówka county; and Białystok, Dobrzyniewo Duże, Supraśl, and Zabłudów, which belong to Białystok county (Figure 1). 

Hajnówka county is located entirely on the North Podlasie Lowland. Characteristic soils for this territory are podzolic, brown, clay-illuvial, and rusty soils. Forests cover approximately 53.28% of Hajnówka county. The most numerous are pine forests, where the basic species is the *Pinus sylvestris* L., then *Picea abies* (L.) H. Karst. Mushroom species were collected from mixed forests. The dominant tree species was *Pinus sylvestris* L. [32].

Bialystok county is located in the central-eastern part of Podlaskie voivodeship, on the Bialystok Upland. The soils in the area, similarly to Hajnówka county, are brown, clay-illuvial, podzolic, and rusty soils. Forests constitute over 39% of the total area of the county. The most common tree species are *Pinus sylvestris* L., *Picea abies* (L.) H. Karst, *Quercus robur* L., *Betula pendula* Roth, *Alnus glutinosa* Gaertn [33]. Mushroom species were collected from mixed forests with the dominant tree species being *Pinus sylvestris* L.

### 2.3. Sample Preparation Procedure 

From the prepared fruiting bodies, samples weighing approximately 100 g from the edible parts (stems and caps) were prepared and dried using the traditional method at 60–70 °C using a mushroom dryer (Model GP-101, MPM Product, Poland) to obtain solid dry matter, and then ground [9]. Until analysis, they were stored without light in polyethylene containers in a desiccator. Then, 0.300 g samples were subjected to mineralization, which was performed in Teflon vessels type DAP100, in 4 cm^3^ concentrated nitric acid (V) using a closed-loop microwave system (Speedwave, Berghof, Germany). The 0.25 cm^3^ of the mineralized samples were diluted in 4.75 cm^3^ deionized water.

### 2.4. Element Determination

The concentrations of the studied elements (Cu, Mn, Se, and Zn) in the diluted edible mushroom mineralization was determined using a plasma ionization mass spectrometer inductively coupled to a quadrupole analyzer (ICP-MS NexION 300D from PerkinElmer, USA). Measurements were taken in the standard mode (separation of ions on 4 quadrupole bars) taking into account the corresponding mathematical corrections. The quadrupole scanning speed (Dwell Time per AMU) was 50 ms. The measurement was taken using a dual detector, enabling simultaneous operation in the pulse and analogue systems. The results obtained in "cps" (counts per second) were converted into the concentrations of the studied elements against the calibration curves. The measurement time of one sample was 35 seconds, with a 15 second delay, to obtain appropriate repeatability of the results. A multi-element calibration was used for the ICP-MS apparatus (Solution-NexION Setup N8145051, PerkinElmer, USA). Two multi-element standards (PerkinElmer Pure Plus USA) were used for the calibration curves: PE N8145054; PE N8145059.

### 2.5. Accuracy Check of the Element Determination Method

The accuracy check of the applied methods of element determination was performed on certified reference materials: Corn Flour INCT-CF-3 and Tea leaves INCT-TL-1, Institute of Nuclear Chemistry and Technology, Warsaw, Poland. In order to check the recovery, a control sample (multi-element standard) was studied every 10 samples.

For each of the examined elements, detection limits were determined, which were expressed as three times the standard deviation for ten blind samples (3× SD from 10 blind samples). Detection limits for individual elements of Cu, Mn, Se, and Zn were: 3.2 μg/L; 0.09 μg/L; 0.19 μg/L; 4.3 μg/L, respectively.

Repeatability for individual elements, Cu, Mn, Se, and Zn was: 1.4%; 2.4%; 4.7%; 1.2%, respectively. 

### 2.6. Evaluation of the Element Content in the Studied Mushrooms in Relation to Nutrition Standards

To estimate the fulfillment of the demand for particular elements as a result of the consumption of an edible portion of the studied mushroom species by an adult, nutrition standards for recommended dietary allowance (RDA) or adequate intake (AI) were used [34,35].

The available literature data show that the average portion of fresh mushrooms consumed by Polish consumers is 100 g [36,37]. Thus, the content of the analyzed elements in 100 g of dry matter was calculated to 100 g of fresh mushrooms and compared with Polish nutrition norms for these elements at the level of the recommended daily allowance (RDA) for an adult [34]. Due to the lack of RDA standards for Mn, the content of this element in a portion was compared with the European standards at the level of adequate intake (AI) [35].

The RDA for individual elements: for Zn, 8 mg/day for women and 11 mg/day for men; for Cu, 0.9 mg/day for women and men; and for Se, 0.055 mg/day for women and men [34]. In the case of Mn, the potential intake of this element in a portion of mushrooms was compared with AI, which is 1.8 mg/day for women and 2.3 mg/day for men [35].

### 2.7. Assessment of the Maximum Permitted Intake Levels

The Cu, Mn, Se, and Zn content in 100 g FM of the studied mushroom species was compared to the upper intake levels (UL). UL values for individual elements are: Cu—5 mg/day, Mn—11 mg/day, Se—0.3 mg/day, and Zn—25 mg/day [38].

The provisional maximum tolerable daily intake (PMTDI) and the provisional maximum tolerable weekly intake (PMTWI) limits are only determined for elements belonging to toxic metals, which include: mercury (Hg), cadmium (Cd), lead (Pb), arsenic (As), copper (Cu), zinc (Zn), and tin (Sn). PMTDI values can be used to assess the exposure to a given element, resulting from the consumption of a food product in which a given element occurs in its natural quantity or as a pollutant [39]. PMTDI limits are expressed in mg/kg of body mass and for Cu are 0.50 mg/kg b.m./day and for Zn 1.0 mg/kg b.m./day [40,41].

In our work, we estimated the percentage share of a given element collected from a 100 g portion of fresh mushrooms in the implementation of PMTDI by an adult of an average body weight of 70 kg [42].

### 2.8. Statistical Analysis

In the statistical analysis, normal distribution was verified using the Lilliefors test (an adaptation of the Kolmogorov–Smirnov test) and the Shapiro–Wilk test. We found no normal distribution of the analyzed quantitative variables. Thus, when comparing them, we used the non-parametric Mann–Whitney U test for two groups. In the case of many groups of variables, we used the nonparametric ANOVA Kruskal–Wallis test with post-hoc analysis of multiple comparisons of mean ranks for all samples. Spearman's rank correlation coefficient was also determined. Results with a level of *P* < 0.05 were considered statistically significant. The computer package Statistica 10.0 (StatSoft, Inc., Tulsa, OK, USA) was used in the calculations.

## 3. Results and Discussion

### 3.1. Evaluation of Moisture Content and the Total Content of the Studied Elements

In the studied mushroom species, the water content was determined, which ranged from 82% (*M. procera*) to approximately 95% (*S. grevillei*) in wild mushrooms and from approximately 91% to approximately 92% in cultivated mushrooms (Table 1). Similar water content in the fruiting bodies of mushrooms was also reported by other authors [10,43,44]. The water content of mushrooms depends on the species, the growing conditions, and the maturity of the fruiting body. For example, the percentage of water in the fruiting body of *I. badia* may vary in the range of 84–91% [45] and in *M. procera* 91–96% [44].

In the studied mushroom species, the total content of the assessed elements was calculated (Table 2). In all the studied mushroom fruiting bodies, we found the lowest selenium content (median 0.37 μg/g DM, mean 1.47 μg/g DM) and the highest Zn content (median 101 μg/g DM, mean 107 μg/g DM). Other authors’ results also indicate a dominant Zn content in the fruiting bodies of mushrooms compared with other elements [46,47].

The lowest content of the analyzed total micronutrients among the wild mushrooms was in *A.mellea*, *S. granulatus*, *L. scabrum*, and *S.grevillei*, while the highest was in *X. chrysenteron* and *M. procera* (Table 3). 

The high content of total elements in *X. chrysenteron* may result from the high zinc content in its fruiting bodies (Table 1), while the high total element content in *M. procera* may be related to the high level of Cu found in the fruiting bodies of this species (Table 1). According to other authors, both *X. chrysenteron* [48,49] and *M. procera* [50] show cumulative properties in relation to the studied elements.

From the cultivated mushrooms, the highest total content of the studied elements was in *A. bisporus* (Table 3). Our results confirm the observations of Rzymski et al., who found the accumulation abilities of this species in relation to elements such as Cu, Se, and Zn [51].

In general, mushrooms are abundant in minerals. Therefore, studies on the inter-relationship between minerals as well as correlation studies between minerals in mushrooms and between minerals in soil have been conducted [49,52]. For certain species of mushrooms, this may be affected by accumulating abilities for some minerals [53].

The content of elements in mushrooms depends on the content of elements in the soil [54]. Wild mushrooms can adapt to small amounts of elements in the soil, e.g., Se, thanks to an ability to form specific hardly soluble inorganic compounds (HgSe) or metalorganic complexes of Se. On the other hand, some mushroom species can adapt to high concentrations of toxic metals in the soil due to specific peptides which bind elements, thereby reducing their toxicity [55].

Both mycorrhizal and saprobic mushrooms secrete enzymes and organic acids to change soil pH and to effectively absorb inorganic compounds [56]. Moreover, certain species of mushrooms have a species-specific affinity for metals. *Russula atropurpurea* is an effective Zn-absorbing species due to special metal-binding proteins functionally related to MTs (metallothionein) [57,58,59]. 

In this study, we found a weak, negative correlation between Se and Mn content (Table 4). Se and Mn have similar properties and chemical behavior. As the other studies show, mushrooms may discriminate similar elements [26]. This is most likely due to the high competitive sorption of metals from the soil or substrate, an effect observed in bio-enriched mushrooms [60]. The higher ability of mushrooms to accumulate Se may be due to the presence of sulfuric amino acids [61]. Selenomethionine is the major compound, among selenocompounds, found in mushrooms [62]. Moreover, some mushrooms have the ability to accumulate Se, while most of them exclude Mn [26,63,64].

### 3.2. Evaluation of Cu Content in the Studied Mushrooms

The Cu content in wild mushrooms ranged in our study from 10.6 μg/g DM (0.09 mg/100 g FM) in *L. deliciosus* to 123 μg/g DM (1.83 mg/100 g FM) in *M. procera* (Table 1). Other Polish studies indicated a similar range for this element, from approximately 10 to 200 μg/g DM [26,63,65,66]. In our research, the Cu content in wild mushrooms was similar to some reports from abroad—12–181 μg/g DM [67], 10.3–145 μg/g DM [68], and 13.4–50.6 μg/g DM [69]. In a Greek study, the Cu content of mushrooms from the natural environment ranged from 7.38 to 75.1 μg/g DM [43]. However, in species corresponding to those studied in our study, including *C. cibarius* and *A. mellea*, the Cu content was approximately two times lower [43]. Both in our research and in the above studies, the mushrooms came from areas free of industrial pollution [65].

The Cu content of mushrooms is to a large extent correlated with the pollution of the region from which the samples are obtained. Some authors point to a large accumulation of Cu in mushrooms from areas close to Cu smelters [70,71]. The content of this element can then reach values of above 500 μg/g DM [64]. We found that the average Cu content in mushroom fruiting bodies was approximately fourteen times lower compared with its content in mushrooms from the reports of Svoboda et al. [70] and Collin-Hansen et al. [71]. Increased Cu content in mushrooms from the above studies may result from soil contamination from which the samples were obtained [70,71]. The earlier study reported on the copper content in the top layer of forest soils near the studied area that ranged from 3.79–15.9 µg/g [72], and 0.48–2.7 [73], while the average copper content of European soils is 17.3 ± 19.0 µg/g [54]. For comparison, soils from contaminated areas, such as the wooded zone surrounding the copper smelter in south-western Poland, may contain even 2380 µg/g [74].

The significantly lower Cu content obtained in fruiting bodies of mushrooms in our study, compared with the above results [70,71], confirms the lack of pollution of the area from which they were obtained. The Cu content recorded in fruiting bodies of mushrooms with strong accumulation properties, from areas free from pollution, ranged from 100 to 300 μg/g DM [75]. According to Soylak et al., such Cu content in mushroom fruiting bodies does not threaten the health of consumers [69]. Some *Agaricus* species and *M. procera* species are among the mushrooms that accumulate Cu [76,77,78]. Some publications have reported that mushrooms characterized by a higher content of Cu (accumulating species) can be a good source of this micronutrient in the diet compared with products of plant origin [76]. 

In our research, we found the highest Cu content in *M. procera* (Table 1). The literature data [64,79,80,81,82] describing the Cu content in mushroom species we investigated (*B. edulis, I. badia, B. subtomentosus, L. scabrum, S. grevillei*, and *M. procera*) also indicated the highest content of this element in the fruiting bodies of *M. procera*. The much higher Cu content in *M. procera*, compared with other species, indicates its ability to accumulate this element. Other authors also indicated the accumulation properties of *M. procera* in relation to Cu [26,76,83]. On the other hand, much lower Cu content in *M. procera* was presented by a Turkish study, in which a greater ability to accumulate this element was demonstrated in *S. granulatus* [12]. In addition, higher Cu content was observed in caps than in the stems, which may result from an increased accumulation of Cu in mushroom hymenophores [52,84].

We found that cultivated mushrooms were characterized by a wide range of Cu content (from 7.3 μg/g DM in *L. edodes* to 40.8 μg/g DM in *A. bisporus*) (Table 1). Cultivated mushrooms from a Finnish study were characterized by a similar content of this element (5.2–35.0 μg/g DM) [85]. The above results may suggest the Cu accumulation ability of double-spore mushrooms. However, a Turkish study involving enriching the substrate with various elements, including Cu, with the aim of determining the accumulation abilities of mushrooms, did not confirm this observation [86].

### 3.3. Evaluation of Manganese Content in Edible Mushrooms

The Mn content of European soils is variable. The average Mn content of European soils is 0.08 ± 0.07 µg/g [54], while the content of Mn from Białowieska Forest (place near our research area) is 100 ± 75 µg/g [73].

We determined the highest Mn content among wild-growing mushrooms in *C. caperatus* (41.0 μg/g DM, 0.3 mg/100g FM). The Mn content in the remaining mushrooms ranged from 12.2 μg/g DM (0.08 mg/100 g FM) in *S. grevillei* to 33.7 μg/g DM (0.27 mg/100 g FM) in *R. heterophylla* (Table 1). In other authors’ papers, the Mn content in wild mushrooms obtained from forest areas throughout Poland was in a similar range (8.74–66.9 μg/g DM) [65]. For example, in the present study, *B. edulis* had only 30% less Mn compared with the research of Falandysz et al., who evaluated the content of this element in *B. edulis* from the Swietokrzyska Forest. In addition, Falandysz et al., based on an analysis of soil and fruiting bodies, excluded the ability of Mn accumulation by the fruiting bodies of this species (bioconcentration factor—BCF < 1) [64]. Referring the results of our research to the results of Rudawska et al., we found that the Mn content was similar in the case of: *L. deliciosus*, *L. aurantiacum*, *S. luteus*, *I. badia*, and *X. chrysenteron* [87].

The content of Mn in the *I. badia*, *B. edulis*, and *C. cibarius* species in this paper was significantly higher compared with analogous species picked in the vicinity of Lodz (from 0.1 to 0.5 μg/g DM) [88]. However, in wild mushrooms from other regions of Europe and in the world, the Mn content ranged from 14 μg/g s.m. up to 145 μg/g DM in whole mushroom fruiting bodies [89], from 12.5 to 29.8 mg/kg DM in the caps, and from 13.3 to 103.9 mg/kg DM in the stems [90].

The Mn content in *A. mellea* and *C. cibarius* in our study was more than twice lower compared with a Turkish study [91], whereas in the case of *X. chrysenteron*, it was in line with other Turkish reports [92]. *C. cibarius* was characterized by a 30% higher content of this element compared to a report from Greece [16]. The Mn content in *B. edulis* was approximately twice as high compared to a Chinese study, while in *R. vinosa* fruiting bodies did not differ significantly [93].

In the present paper, the Mn content in cultivated mushrooms ranged from 5.91 μg/g DM (0.05 mg/100g FM) in *A. bisporus* to 27.1 μg/g DM (0.24 mg/100 g FM) in *L. edodes* (Table 1). The obtained values were in line with the results of a Hungarian study, in which the content of twenty-three elements, including Mn, was analyzed in three species of cultivated mushrooms: *A. bisporus*, *P. ostreatus*, and *L. edodes* [94]. The obtained Mn content in the *A. bisporus* fruiting bodies in the present study was lower than the range obtained for this element in the samples from Australian common mushroom farms [95].

Isildak et al. [96] observed much higher values of Mn in wild *A. bisporus*, *P. ostreatus* and *L. edodes* species compared with their cultivated counterparts. For example, the fruiting bodies of *A. bisporus* obtained from natural conditions in the Tokat province in Turkey were characterized by much higher Mn content (25.9 μg/g DM) compared with the fruiting bodies of the same cultivated species [96]. However, compared to the study of Gençcelep et al. [12] the Mn content in the fruiting bodies of *P. ostreatus* was more than twice lower (Table 1). Studies by other authors showed that mushrooms did not exhibit accumulation properties in relation to Mn. In most literature data, the bioconcentration factor (BCF) was less than one (BCF < 1) [26,63,64].

### 3.4. Evaluation of Se Content in Edible Mushrooms

The concentration and distribution of selenium in the Earth's crust is uneven. A significant part of Europe, including Poland, is an area poor in this element. The natural concentration of Se in soil ranges from 0.1 to 2.0 µg/g DM. The average concentration of selenium in top layers of soils worldwide is below 0.5 µg/g DM [97]. The mean Se content in Polish soils is 0.27 µg/g DM, in the soils of southern Poland ranges from 0.06–0.81 μg/g DM, and in north-eastern Poland 0.11-1.57 µg/g DM [98].

In the present study, we found that the Se content in mushrooms ranged from 0.13 μg/g DM (0.001 mg/100 g FM) in *A. mellea* to 13.3 μg/g DM (0.12 mg/100 g FM) in *B. edulis* (Table 1). The Se content in *B. edulis* in our study was more than twice lower compared with the study of Jarzyńska et al. [99], who assessed Se from fruiting bodies from mountainous areas (Sudety Mountains). We found that the average Se content in *B. edulis* was significantly higher compared with other species of wild and cultivated mushrooms, which indicates the accumulating properties of this species (Table 1, Table 2). Authors assessing the presence of Se in other species from the *Boletaceae* family did not find high levels of this element [17], which may indicate that Se can only be accumulated by *Boletus* fruiting bodies. The *Boletus* species that accumulate Se also include *B. pinicola*, *B. aestivalis* and *B. appendiculus* [100]. The accumulation properties of *Boletus* mushrooms were also confirmed in a Chinese study, in which the highest Se content was found in *B. chrysenteron* (now *X. chrysenteron*) (approximately 19 μg/g DM), and was almost seventy times higher than in the soil from which the fruiting bodies were obtained [101].

Of the wild edible mushrooms studied so far, only a few had accumulation properties in relation to Se. The species with the highest average Se content was *A. pes-caprae* (approximately 200 μg/g DM) [100], while the highest recorded Se content in this species reached 370 μg/g DM [102]. 

The most common edible species accumulating Se was *B. edulis*. The average content of this element in its fruiting bodies was 20 μg/g DM [100]. However, the highest recorded content was 70 μg/g DM [100]. The high Se content in *Boletus* was also observed in Portuguese [17], Italian [103], and Czech studies [104].

Among the cultivated mushroom species, the highest Se content was found in *A. bisporus*. It was higher compared with its content in other cultivated mushroom species, *P. ostreatus* and *L. edodes* (Table 1). In the case of *P. ostreatus* and *L. edodes*, the Se content was more than thirty times and twenty times lower, respectively (Table 1). A similar tendency was observed in a Portuguese study [17].

In the present study, the Se content in *A. bisporus* was within the upper limits of the range found for mushrooms in other regions of the world (0.2–6.6 μg/g DM) [78,100,105]. Whereas, Se content in *P. ostreatus* and *L. edodes* was within the lower limits of the range found by other authors (0.12–3.4 μg/g DM) [17,106].

Until recently, Se in mushrooms was considered to be an element with very low bioavailability for humans and animals. However, in vivo studies confirmed the possibility of using mushrooms accumulating Se (occurring in the natural environment and cultivated on a substrate enriched with Se compounds) as a functional food [107,108]. Research from India conducted on *P. florida* showed that mushrooms of this species grown on a Se-rich substrate contained eight hundred times higher content of this element in relation to control samples grown on a normal substrate. Furthermore, after simulated gastrointestinal digestion in vitro, 75% of Se was extracted from the sample, with almost one hundred percent in the form of well-absorbed selenomethionine [61].

### 3.5. Evaluation of Zn Content in Edible Mushrooms

The average Zn content of European soils was 68 ± 141µg/g [54], while the content of Zn from the soils of Białowieska Forest (place near our research area) was 8.3 ± 4.4 µg/g [26].

From the analyzed components, Zn is the dominant element in the studied mushrooms (Table 1, Table 3). This may be due to its accumulation in the fruiting bodies of most species [79,109]. The content of Zn in the studied wild mushrooms was in a range of 68.3 μg/g DM (0.77 mg/100 g FM) in *A. mellea* to 184 μg/g DM (1.10 mg/100 g FM) in *X. chrysenteron* (Table 1). Similar results were reported by other authors, who found the highest content of Zn in the fruiting bodies of *X. chrysenteron* [65] and lower content in the fruiting bodies of *A. mellea* from northern Poland [109]. Almost twice as much Zn was noted for *A. mellea* fruiting bodies from the southern regions of Poland (40.9 μg/g DM) [110]. 

Polish research conducted on several dozen fruiting bodies of *I. badia* found large variation in the Zn content in this species, even within one picking location (64–240 μg/g DM) [80]. They also indicated the tendency to accumulate Zn by the fruiting bodies of this species. In our research, the Zn content of *I. badia* fruiting bodies was in the range cited by Kojta et al. [80] and was 144 μg/g DM (Table 1). A slightly lower content of this element in *I. badia* was found by Adamiak et al. (126 μg/g DM) [46], while slightly higher by Reczyński et al. (172 μg/g DM) [24].

We also found high Zn content (170 μg/g DM) in *B. edulis* (Table 1). Similar values were obtained by Falandysz and Borovička [26], and slightly lower by Adamiak et al. (137 μg/g DM) [46].

However, in the case of *L. rufum*, our results were lower (Table 1, 115 μg/g DM) compared with those found by Adamiak et al. (186 g/100 g DM) [46].

The Zn content in *C. cibarius* was on average 121 μg/g DM (0.95 mg/100 g FM) (Table 1) and was in the range presented by Drewnowska and Falandysz [111].

In our study, Zn content (Table 1) in mushroom fruiting bodies was in the ranges obtained for this element in mushrooms from outside of Poland (25–200 μg/g DM) [67,78,112]. A study from Spain indicated a high capacity of Zn accumulation by the fruiting bodies of *L. deliciosus* [52]. We made similar observations in our study, in which *L. deliciosus* was in the group of mushrooms with the highest Zn content (Table 1). Compared with the study of Alonso et al. [52], *A. bisporus*, *P. ostreatus*, *B. edulis*, *C. cibarius*, *L. aurantiacum*, *M. procera*, *T. equestre*, and *X. chrysenteron* were characterized by slightly higher Zn content, while slightly lower Zn content was noted in *T. portentosum* and *I. badia* [52].

Compared with a study from Turkey [113], the Zn content in *A. bisporus* was slightly higher (Table 1), and in the case of *Imleria badia*, it was more than three times higher (Table 1). With regard to the research of other Turkish authors, the Zn content found in *A. mellea* and *C. cibarius* was slightly lower, but the tendency in Zn content among different mushroom species was preserved (*C. cibarius* > *A. mellea*) [114].

In our research, the Zn content in cultivated mushrooms of the: *A. bisporus*, *P. ostreatus*, and *L. edodes* species (Table 1) was similar to the content of this element in analogous species in a Chinese study [89]. In the case of *L. edodes*, the mean values obtained in both studies were approximately 60 μg/g DM. In *A. bisporus*, the average Zn content in our study was 22% lower, while in the case of *P. ostreatus*, it was 33% higher [89]. In a Turkish study conducted on thirty mushroom species, an ability to accumulate Zn in *P. ostreatus* was found [12].

The higher content of Zn in wild mushrooms may be related to the higher content of this element in the soil. As shown in previous reports, mushrooms show a high capacity for accumulating Zn [79].

### 3.6. Microelement Contents in Wild Mushrooms vs. Commercial Mushrooms

Despite the fact that previous soil research in the forests in the Podlasie region did not indicate any elevated concentrations of the studied microelements [72,115], we found statistically higher Cu and Zn content in wild mushrooms compared with cultivated mushrooms (Table 5). 

The same trend, although not significant, was observed for Mn. The higher Cu, Mn, and Zn content in wild mushrooms may result from several environmental factors: a higher content of these elements in the soil compared with the soil used for growing commercial mushrooms; favorable pH; the presence of organic matter, environmental pollution, and from the several mushroom factors, such as favorable mushroom structure, biochemical composition, decomposing activity (higher in saprophite species), and the development of hyphe [52].

Cu, Mn, and Zn are the main components of the mineral fractions of soil, therefore are accessible for wild mushrooms [116]. Moreover, forest soils are characterized by a high content of organic matter and by an acidic pH, which foster the formation of mobile mineral-organic complexes [115]. In addition, higher Cu, Mn, and Zn content in wild mushrooms may be explained by the presence of their network of hyphae in the upper layer of the soil. The hyphe of wild mushrooms can be spread over several square meters, which allows to absob crucial minerals and water [52,58,66]. The content of elements in cultivated mushrooms could be higher if the substrates on which they grow are enriched with elements and other subtances supporting absorption [117].

### 3.7. Daily Intake of the Assessed Elements

The percentages of the recommended daily allowance of the elements assessed in a 100 g portion of mushrooms are presented in Table 6.

We found that *M. procera* is the best source of Cu from the studied wild-growing mushrooms. Consuming a portion (100 g FM) of *M. procera* fruiting bodies will provide 203% of the RDA for Cu in the case of women and men (Table 6).

In contrast, Rajkowska-Mysliwiec et al. [47] reported that consuming a 100 g portion of fresh mushrooms by adults covers the daily Cu requirement at approximately 40% in the case of *B. edulis*, 30% for *L. scabrum*, 56% for *C. cibarius*, and 50% for *I. badia*. Chudzyński and Falandysz [79] estimated the RDA percentage for this element as a result of consuming *S. grevillei* at 24–37%, depending on the mushroom picking location. 

In an Australian study, the daily recommended allowance for Cu realized by *A. bisporus* was estimated at 35% [118]. We obtained a comparable result (Table 6). 

In our research, the implementation of adequate intake (AI) for Mn can be satisfied to the greatest extent as a result of consuming a portion (100 g FM) of *S. bovinus* (17.4% for women and 13.6% for men), while consuming *S. grevillei* will not meet even 1% of the demand for this element (Table 6). Whereas, in the study by Chudzyński et al., consuming a portion (300 g FM) of *S. grevillei* would enable implementing 35–87% of AI [79]. Rajkowska-Mysliwiec et al. showed that consuming a portion of wild mushrooms (100 g FM) by adults may cover 2.4–11% of the demand for Mn. From the studied species (*B. edulis, L. scabrum, C. cibarius*, and *I. badia*), the best source of Mn turned out to be the fruiting bodies of *C. cibarius* [47]. 

Of the cultivated mushrooms, the most Mn was in *L. edodes* (10.4–13.3%) (Table 6). The fruiting bodies of *A. bisporus* were characterized by a much lower standard implementation percentage, which is confirmed by a study in which consuming a portion (100 g FM) of *A. bisporus* ensured only 2–4% AI [95].

Taking into account our study results, we found that the best source of Se in the diet can be *B. edulis* and *M. procera* from wild mushrooms (Table 1), while from cultivated mushrooms, *A. bisporus* (Table 1). Consuming a portion of fresh mushrooms (100 g) can ensure the implementation of the nutritional standard at 210% in the case of *B. edulis*, and 78% in the case of *M. procera*. Thus, it can be assumed that already approximately 50 g FM of *B. edulis* can cover the daily requirement for this element for an adult. Costa-Silva et al. also indicated the high RDA percentage of Se in *Boletus* mushrooms [17]. In the study of Costa-Silva et al., the standard implementation as a result of consuming 100 g FM of mushrooms was estimated in the case of *B. reticulatus* at 882%; in the case of *B. pinophilus* at 362%, and *B. edulis* 271% [17].

We estimated that consuming 100 g FM of *A. bisporus* fruiting bodies is able to cover the RDA for Se almost 100% (Table 6). *A. bisporus* is classified as a species with Se accumulation properties [61]. An Australian study found that consuming 100 g of *A. bisporus* affects the recommended daily allowance by 17% [118].

The Se content in cultivated mushrooms may increase as a result of enriching the substrate with inorganic Se compounds [119]. For example, *L. edodes* grown on a substrate enriched with sodium selenate may cover the demand for this element for an adult (55 μg/day) when its consumption amounts to approximately 30 g FM [120]. Whereas, da Silva et al. found that consuming 1 g of dried *P. ostreatus* from selenium-enriched substrate ensured that daily intake is 100% [106].

We estimated that from wild mushrooms *T. equestre* was the most able to meet the needs for Zn (21.9% for women and 15.9% for men) (Table 6). In the study of Mleczek et al. [65], involving five species of wild mushrooms: *L. aurantiacum*, *I. badia*, *B. edulis*, *C. cibarius*, *and S. luteus,* it was found that the Zn requirement could be best covered by *L. aurantiacum* (14.5% in the case of men and 20% for women) [65]. However, Rajkowska-Mysliwiec et al., who studied four mushroom species: *B. edulis*, *L. scabrum*, *C. cibarius*, and *I. badia*, found that the need for Zn will be covered to the largest extent (25%) by consuming 100 g FM of *L. scabrum* [47].

We estimated that the percentage intake of RDA norms for Zn as a result of consuming an average portion (100 g FM) of three cultivated mushroom species, *A. bisporus*, *L. edodes*, and *P. ostreatus*, did not differ significantly. Moreover, they were approximately three times lower compared with wild mushrooms (Table 6). In an Australian study, also conducted using cultivated mushrooms, the obtained results were similar [118].

Mushrooms are generally characterized by a high Zn content. It is one of the dominant elements in this group of organisms [24]. Simultaneously, the Zn contained in mushrooms can be a good source of this micronutrient in the diet [121].

### 3.8. Assessment of the Potential Risks Associated with the Consumption of the Studied Wild Mushrooms

We analyzed the toxicological risk of consuming a portion of mushrooms (100 g FM) [25] by an adult weighing 70 kg [37].

The content of individual elements in dry matter was calculated per 100 g FM of fruiting bodies (average portion) and compared with the toxicological limits at upper tolerable intake level UL and provisional maximum tolerable daily intake (PMTDI).

We found that the consumption of 100 g FM of mushrooms with the highest Cu content (*M. procera*, 1.83 mg/100 g FM) will satisfy 36.6% of UL implementation, and, in the case of PMTDI, only 5.23%. Therefore, safe intake (PMTDI) of Cu will be exceeded by a consumer with a body weight of 70 kg in the case of the consumption of more than 1.95 kg of *M. procera* in a day. Analogous results were obtained by authors investigating mushroom fruiting bodies from areas located far away from contaminants [43,69,100,116]. On the other hand, literature reports describing the content of this element in mushrooms obtained from contaminated sites located near Cu smelters show the content of this element at a level that is a risk to the health of consumers [70,71]. In the body, excessive amounts of copper are deposited in the liver, brain, and eye cornea resulting in damage to these organs and cognitive impairment [34].

No toxicological threat was found from the Mn present in the *S. bovinus* and *C. caperatus* fruiting bodies, which were characterized by the highest content of this element. The consumption of 100 g of FM fruiting bodies of these species will affect only approximately 3% of the UL (Table 7). It was estimated that only very high intake, of over 3.5 kg/day, of fresh mushrooms of this species may cause exceeding the UL set for Mn. Excessive long-term manganese exposure can cause neurotoxic symptoms such as muscle pain, fatigue, delayed response to stimuli, and memory problems [34].

We found a high proportion of Se in *B. edulis*, which significantly increased the probability of exceeding the UL, which can be achieved by consuming approximately 0.59 kg FM of fruiting bodies of *B. edulis.* The most characteristic symptoms of chronic selenium poisoning are nail brittleness and loss, increased sweating, and garlic breath. In addition, there may be neurological symptoms such as nervousness, emotional instability, and gastrointestinal disorders [34].

However, the consumption of higher than recommended Se doses may protect from prostate and colon cancer [122,123]. Furthermore, the importance of mushrooms as a source of micronutrients is dependent on the season. It is the highest from late summer through late autumn and Christmas [47]. At present, at least one billion people in the world are at risk for Se deficiency in their diet [90,97,124]. That is why it is very important to look for new ways to enrich the diet with this element in areas where it is lacking. It may be a good idea to encourage people from these areas to consume wild-growing, edible mushrooms characterized by the ability to accumulate this element. *B. edulis*, among others, has such properties (Table 1). 

We found that the UL and the PMTDI determined for Zn were not exceeded. The consumption of a portion of mushrooms (100 g FM) with the highest Zn content found was 92.9% lower than the UL and 97.5% lower than PMTDI (Table 7). It was estimated that intake over 0.4 kg/day of fresh mushrooms may cause exceeding the PMTDI set for Zn.

The usual amounts of zinc content in food do not contribute to excessive Zn intake. However, prolonged intake of high doses of Zn leads to a decrease in immune response and a worsening of copper and iron nutrition [34].

No toxicological risk was identified from the potential consumption of the highest levels of Cu, Mn, Se, and Zn in the studied mushrooms in relation to international standards. In addition, our earlier studies confirmed the safety of mushrooms originating from the GLP region, also in terms of heavy metals contamination, such as cadmium (Cd) and lead (Pb) [125]. Other authors showed that the content of bioelements in mushrooms from unpolluted areas does not pose a toxicological threat and that mushrooms may be a good dietary source of Cu, Mn, Se, and Zn [26,27,53]. In our study, some mushrooms, especially *M. procera, S. bovinus, B. edulis* and *T. equestre*, can be recommended for consumption as good sources of Cu, Mn, Se, and Zn, respectively.

Mushrooms generally show a higher content of essential trace elements compared with other food products, for example vegetables and fruits, but comparable to cereals and nuts [126,127]. Therefore, they can be a good supplementary food to some populations with low dietary mineral intake [128,129,130].

## 4. Conclusions

In this paper, we report on the contents of Cu, Mn, Se, and Zn in 18 species of edible mushrooms from an unpolluted area called the Green Lungs of Poland, which is free of big industry and with a low urbanization rate, with the aim to compare them with commercial mushrooms and make comparisons between the species.

We also studied to what extent mushrooms meet the demand for these elements in adults and whether their consumption poses a toxicological hazard in relation to these elements.

It was shown that the studied wild mushrooms can be a good supplementary source of Cu, Mn, Se, and Zn for adults. Moreover, wild edible mushrooms can be a better source of Cu, Mn, and Zn in comparison with commercially available mushrooms. In particular, mushrooms species such as *M. procera*, *S. bovinus, B. edulis*, and *T. equestre* can be recommended for consumption because of their high content of Cu, Mn, Se, and Zn, respectively.

In relation to dietary recommendations regarding the studied elements, a portion of mushrooms ensures meeting the demand for the analyzed elements within 1–96.4%, with the exception of *B. edulis* and *M. procera*, which can provide Se and Cu, respectively, in quantities exceeding the demand for these elements. At the same time, these mushrooms do not pose a toxicological risk resulting from their consumption. 

By comparing the contents of the studied elements in a fresh portion of mushrooms with the toxicological limits (UL and PMTDI), we found no toxicological risk associated with the consumption of mushrooms with the highest content of Cu, Mn, Se, and Zn for the average adult.

## Figures and Tables

**Table 1:** Microelement content in wild and cultivated edible mushrooms in dry and fresh mass.

No.	Mushroom Species	N	Moisture (%)	Cu µg/g DM	Cu mg/100 g FM	Mn µg/g DM	Mn mg/100 g FM	Se µg/g DM	Se mg/100 g FM	Zn µg/g DM	Zn mg/100 g FM
				X ± SD							
1.	*Agaricus bisporus*	3	91.5–92.5	40.8 ± 10.5	0.33 ± 0.07	5.91 ± 0.63	0.05 ± 0.01	6.17 ± 5.70	0.05 ± 0.05	63.4 ± 19.2	0.51 ± 0.16
2.	*Armillaria mellea*	3	86.6–91.8	23.0 ± 3.8	0.26 ± 0.08	24.7 ± 1.60	0.28 ± 0.07	0.13 ± 0.03	<0.01	68.3 ± 10.6	0.77 ± 0.19
3.	*Boletus edulis*	3	90.7–92.0	19.0 ± 5.0	0.16 ± 0.04	20.1 ± 8.9	0.17 ± 0.07	13.3 ± 5.60	0.12 ± 0.06	170 ± 33.7	1.46 ± 0.27
4.	*Boletus subtomentosus*	3	88.4–92.6	18.5 ± 2.59	0.18 ± 0.06	16.3 ± 7.48	0.15 ± 0.03	0.30 ± 0.07	<0.01	112 ± 17.0	1.08 ± 0.34
5.	*Cantharellus cibarius*	4	91.5–93.2	69.0 ± 3.48	0.54 ± 0.08	32.5 ± 7.51	0.26 ± 0.08	0.26 ± 0.02	<0.01	121 ± 36.9	0.95 ± 0.33
6.	*Cortinarius caperatus*	4	91.2–93.3	57.1 ± 21.5	0.42 ± 0.15	41.0 ± 17.3	0.30 ± 0.12	0.88 ± 0.26	0.01 ± 0.00	90.2 ± 34.4	0.66 ± 0.22
7.	*Imleria badia*	4	89.2–93.6	41.3 ± 9.31	0.36 ± 0.15	27.6 ± 10.4	0.22 ± 0.05	0.28 ± 0.04	<0.01	144 ± 31.6	1.24 ± 0.49
8.	*Lactarius deliciosus*	3	90.8–92.1	10.6 ± 4.28	0.09 ± 0.04	13.0 ± 2.51	0.11 ± 0.01	1.46 ± 0.33	0.01 ± 0.00	129 ± 19.0	1.11 ± 0.18
9.	*Leccinum rufum*	3	90.5–91.5	50.9 ± 5.05	0.47 ± 0.07	15.1 ± 1.91	0.14 ± 0.03	1.17 ± 0.32	0.01 ± 0.00	115 ± 18.3	1.05 ± 0.22
10.	*Leccinum scabrum*	3	83.9–91.8	16.6 ± 3.42	0.21 ± 0.11	13.5 ± 10.4	0.18 ± 0.14	0.58 ± 0.32	0.01 ± 0.00	87.7 ± 24.1	1.10 ± 0.54
11.	*Lentinula edodes*	3	90.9–90.8	7.3 ± 2.41	0.06 ± 0.02	27.1 ± 0.92	0.24 ± 0.01	0.22 ± 0.03	<0.01	61.4 ± 57.8	0.54 ± 0.01
12.	*Macrolepiota procera*	3	82.0–87.1	123.0 ± 34.9	1.83 ± 0.23	16.3 ± 2.11	0.25 ± 0.01	2.93 ± 0.97	0.04 ± 0.01	84.1 ± 4.43	1.28 ± 0.15
13.	*Pleurotus ostreatus*	4	90.9–91.9	14.1 ± 2.50	0.12 ± 0.02	12.9 ± 0.61	0.11 ± 0.01	0.19 ± 0.01	<0.01	72.4 ± 10.1	0.61 ± 0.06
14.	*Russula heterophylla*	3	92.5–94.8	19.9 ± 3.67	0.17 ± 0.09	33.7 ± 0.81	0.27 ± 0.11	0.26 ± 0.03	<0.01	115.9 ± 16.3	0.95 ± 0.52
15.	*Russula vinosa*	3	86.3–90.4	77.2 ± 18.0	0.87 ± 0.34	27.8 ± 8.85	0.29 ± 0.05	0.25 ± 0.06	<0.01	68.9 ± 11.3	0.75 ± 0.09
16.	*Suillus bovinus*	3	82.9–85.7	15.2 ± 3.73	0.24 ± 0.04	19.9 ± 17.2	0.31 ± 0.28	0.95 ± 0.10	0.02 ± 0.00	81.6 ± 28.1	1.28 ± 0.44
17.	*Suillus grevillei*	4	92.4–95.7	29.3 ± 1.41	0.17 ± 0.04	12.2 ± 10.4	0.08 ± 0.09	0.66 ± 0.11	<0.01	93.9 ± 66.1	0.54 ± 0.19
18.	*Suillus luteus*	3	91.3–94.2	17.8 ± 3.48	0.13 ± 0.04	20.3 ± 7.5	0.15 ± 0.06	1.64 ± 0.82	0.01 ± 0.01	101 ± 15.7	0.71 ± 0.17
19	*Tricholoma equestre*	3	86.3–92.1	25.2 ± 15.6	0.27 ± 0.11	15.9 ± 3.05	0.16 ± 0.05	0.43 ± 0.06	<0.01	176 ± 25.4	1.75 ± 0.27
20.	*Tricholoma portentosum*	3	91.6–93.4	15.0 ± 1.34	0.11 ± 0.02	14.9 ± 4.49	0.11 ± 0.03	0.29 ± 0.04	<0.01	112.6 ± 4.64	0.85 ± 0.07
21	*Xerocomellus chrysenteron*	3	93.4–94.4	36.8 ± 7.56	0.22 ± 0.05	21.7 ± 6.43	0.13 ± 0.03	0.26 ± 0.03	<0.01	184 ± 35.0	1.10 ± 0.14

N–number of samples; Cu–copper, Mn–manganese; Se–selenium; Zn–zinc; DM–dry mass; FM–fresh mass; X–mean value; SD–standard deviation.

**Table 2:** Average and median content of Cu, Mn, Se and Zn in the studied mushrooms (μg/g DM).

No.	Microelements	N	X ± SD	Me	Q1	Q3
			μg/g DM			
3.	Cu	68	**35.2 ± 28.5**	**25.4**	15.6	45.5
2.	Mn	68	20.8 ± 11.1	18.3	12.8	27.5
4.	Se	68	1.5 ± 3.2	0.37	0.25	1.07
1.	Zn	68	107 ± 40.2	101	75.0	130

N–number of samples; Zn–zinc; Mn–manganese; Cu–copper; Se–selenium; DM–dry mass; X–mean value; SD–standard deviation; Me–median; Q1–first quartile; Q3–third quartile.

**Table 3:** Total microelement content in the dry and fresh mass of the studied mushrooms.

No.	Mushroom Species	Cu + Mn + Se + Zn µg/g DM	Cu + Mn + Se + Zn mg/100 g FM
18.	*A. bisporus (white)*	116	0.9
19.	*A. mellea*	116	1.3
3.	*B. edulis*	223	1.9
12.	*B. subtomentosus*	147	1.4
7.	*C. caperatus*	189	1.4
4.	*C. cibarius*	222	1.7
6.	*I. badia*	213	1.8
11.	*L. deliciosus*	154	1.3
8.	*L. scabrum*	182	1.7
17.	*L. scabrum*	118	1.5
20.	*L. edodes*	95.9	0.9
2.	*M. procera*	226	3.4
21.	*P. ostreatus*	99.5	0.8
9.	*R. heterophylla*	170	1.4
10.	*R. vinosa*	174	1.9
16.	*S. granulatus*	118	1.8
15.	*S. grevillei*	136	0.8
14.	*S. luteus*	141	1.0
5.	*T. equestre*	217	2.2
13.	*T. portentosum*	143	1.1
1.	*X. chrysenteron*	243	1.5

Cu–copper; Mn–manganese; Se–selenium; Zn–zinc; DM–dry mass; FM–fresh mass.

**Table 4:** Correlation coefficients between the content of Cu, Mn, Se, and Zn in the studied mushrooms.

*N* = 68 µg/g DM	Cu	Mn	Se	Zn
**Cu**	─	─	─	─
**Mn**	r = 0.18 *p* = 0.14	─	─	─
**Se**	r = 0.12 *p* = 0.33	r = −0.27 *p* = 0.03	─	─
**Zn**	r = 0.10 *p* = 0.41	r = 0.13 *p* = 0.29	r = 0.18 *p* = 0.15	─

N–number of sample; DM–dry mass.

**Table 5:** Cu, Mn, Se, and Zn content in the studied mushrooms depending on the growth environment.

No.	Microelements	Wild Mushrooms *N* = 58		Commercial Mushrooms *N* = 10		*p*
		µg/g DM				
**1.**	**Cu**	**Q1**	**Q3**	**Q1**	**Q3**	─
		18.9	49.7	10.1	29.5	─
		**Me**				─
		27.6		15.0		**0.02**
		**X ± SD**				─
		37.8 ± 29.5		20.2 ± 15.5		─
**2.**	**Mn**	**Q1**	**Q3**	**Q1**	**Q3**	─
		12.9	29.0	6.62	26.1	─
		**Me**				─
		18.9		12.9		0.06
		**X±SD**				─
		21.8 ± 11.2		15.1 ± 0.72		─
**3.**	**Se**	**Q1**	**Q3**	**Q1**	**Q3**	─
		0.26	1.11	0.19	1.03	─
		**Me**				─
		0.46		0.22		0.07
		**X ± SD**				─
		1.38 ± 3.08		1.99 ± 3.99		─
**4.**	**Zn**	**Q1**	**Q3**	**Q1**	**Q3**	─
		83.3	13.7	61.2	74.9	─
		**Me**				─
		110		69.21		**<0.01**
		**X ± SD**				─
		114 ± 39.3		66.6 ± 11.8		─

N–number of samples; Cu–copper; Mn–manganese; Se–selenium; Zn–zinc.

**Table 6:** Percentage implementation of nutrition standards for Cu, Mn, Se, and Zn.

No.	Fresh Mushrooms	Cu		Mn		Se	Zn	
		RDA (%)		AI (%)		RDA (%)	RDA (%)	
		W + M	W		M	W + M	W	M
1.	*A. bisporus (white)*	36.3	2.7		2.1	92.7	6.4	4.6
2.	*A. mellea*	29.0	15.5		12.1	1.8	9.6	7.0
3.	*B. edulis*	18.1	9.5		7.4	**211**	18.2	13.3
4.	*B. subtomentosus*	20.1	8.1		6.3	5.5	13.5	9.8
5.	*C. cibarius*	60.2	14.3		11.2	3.6	11.8	8.6
6.	*C. caperatus*	46.8	16.4		12.9	12.7	8.3	6.0
7.	*I. badia*	39.8	12.2		9.6	3.6	15.5	11.3
8.	*L. deliciosus*	10.4	6.2		4.8	23.6	13.9	10.1
9	*L. rufum*	51.8	7.7		6.0	20.0	13.2	9.6
10.	*L. scabrum*	23.6	10.0		7.8	14.5	13.7	10.0
11.	*L. edodes*	7.1	13.3		10.4	3.6	6.8	4.9
12.	*M. procera*	**203.3**	13.6		10.7	78.2	16.0	11.6
13.	*P. ostreatus*	13.2	6.1		4.8	3.6	7.7	5.6
14.	*R. heterophylla*	18.4	14.8		11.6	3.6	11.9	8.7
15.	*R. vinosa*	96.4	16.3		12.8	5.5	9.4	6.8
16.	*S. bovinus*	26.3	17.4		13.6	27.3	16.0	11.6
17.	*S. grevillei*	19.0	1		1	7.3	6.8	4.9
18.	*S. luteus*	13.9	8.1		6.3	21.8	8.8	6.4
29.	*T. equestre*	29.4	9.0		7.0	3.6	**21.9**	**15.9**
20.	*T. portentosum*	12.6	6.2		4.9	7.3	10.6	7.7
21.	*X. chrysenteron*	24.8	7.2		5.6	3.6	13.8	10.0

Cu–copper; Mn–manganese; Se–selenium; Zn–zinc; RDA–Recommended Dietary Allowance; AI–Adequate Intake; W–women; M–men.

**Table 7:** Percentage of the elements contained in an edible portion (100 g FM) of the studied mushrooms in the implementation of the upper tolerable intake level (UL) and the provisional maximum tolerable daily intake (PMTDI) for Cu and Zn by a person with an average body weight of 70 kg.

No.	Mushrooms	Cu		Mn	Se	Zn	
		UL (%)	PMTDI (%) for 70 kg b.w.	UL (%)	UL (%)	UL (%)	PMTDI (%) for 70 kg b.w.
1.	*A. bisporus (white)*	6.54	0.93	0.44	17.0	2.04	0.73
2.	*A. mellea*	5.22	0.74	2.54	0.33	3.06	1.09
3.	*B. edulis*	3.26	0.46	1.55	**38.7**	5.84	2.08
4.	*B. subtomentosus*	3.62	0.52	1.33	1.00	4.32	1.54
5.	*C. cibarius*	10.8	1.55	2.35	0.67	3.78	1.35
6.	*C. caperatus*	8.42	1.20	2.69	2.33	2.65	0.94
7.	*I. badia*	7.16	1.02	2.00	0.67	4.96	1.77
8.	*L. deliciosus*	1.88	0.27	1.01	4.33	4.45	1.58
9.	*L. rufum*	9.32	1.33	1.25	3.67	4.21	1.50
10.	*L. scabrum*	4.24	0.60	1.64	2.67	4.39	1.56
11.	*L. edodes*	1.28	0.18	2.18	0.67	2.17	0.78
12.	*M. procera*	**36.6**	**5.23**	2.23	14.33	5.12	1.83
13.	*P. ostreatus*	2.38	0.34	1.00	0.67	2.46	0.88
14.	*R. heterophylla*	3.32	0.47	2.42	0.67	3.81	1.36
15.	*R. vinosa*	17.4	2.48	2.67	1.00	2.99	1.06
16.	*S. bovinus*	4.74	0.68	**2.85**	5.00	5.12	1.83
17.	*S. grevillei*	3.42	0.49	0.72	1.33	2.16	0.77
18.	*S. luteus*	2.50	0.35	1.32	4.00	2.83	1.01
19.	*T. equestre*	5.30	0.76	1.47	0.67	**7.02**	**2.50**
20.	*T. portentosum*	2.26	1.00	1.02	1.33	3.39	1.21
21.	*X. chrysenteron*	4.46	0.64	1.17	0.67	4.42	1.57

b.w.–body weight; Cu copper; Mn manganese; Se-selenium; Zn–zinc; PMTDI–Provisional Maximum Tolerable Daily Intake; UL–Tolerable Upper Intake Level; W–women; M–men.
